# Transcriptomic analysis reveals the *GRAS* family genes respond to gibberellin in *Salvia miltiorrhiza* hairy roots

**DOI:** 10.1186/s12864-020-07119-3

**Published:** 2020-10-27

**Authors:** Wenrui Li, Chuangfeng Liu, Jingling Liu, Zhenqing Bai, Zongsuo Liang

**Affiliations:** 1 Institute of Soil and Water Conservation, Chinese Academy of Sciences and Ministry of Water Resources, Yangling, 712100 China; 2 School of Food and Biological Engineering, Shaanxi University of Science and Technology, Xi’an, 710021 China; 3 College of Life Sciences, Northwest A&F University, Yangling, 712100 China; 4 College of Life Sciences and Medicine, The Key Laboratory of Plant Secondary Metabolism and Regulation of Zhejiang Province, Zhejiang Sci-Tech University, Hangzhou, 310018 China

**Keywords:** Transcriptome, GRAS family, Gibberellin, *Salvia miltiorrhiza* hairy roots, Secondary metabolism

## Abstract

**Background:**

*Salvia miltiorrhiza* is one of the most important traditional Chinese medicinal plants with high medicinal value. Gibberellins are growth-promoting phytohormones that regulate numerous growth and developmental processes in plants. However, their role on the secondary metabolism regulation has not been investigated.

**Results:**

In this study, we found that gibberellic acid (GA) can promote hairy roots growth and increase the contents of tanshinones and phenolic acids. Transcriptomic sequencing revealed that many genes involved in the secondary metabolism pathway were the GA-responsive. After further analysis of GA signaling pathway genes, which their expression profiles have significantly changed, it was found that the GRAS transcription factor family had a significant response to GA. We identified 35 *SmGRAS* genes in *S. miltiorrhiza*, which can be divided into 10 subfamilies. Thereafter, members of the same subfamily showed similar conserved motifs and gene structures, suggesting possible conserved functions.

**Conclusions:**

Most *SmGRAS* genes were significantly responsive to GA, indicating that they may play an important role in the GA signaling pathway, also participating in the GA regulation of root growth and secondary metabolism in *S. miltiorrhiza*.

## Background

*Salvia miltiorrhiza* Bunge (Danshen) is a well-known traditional Chinese medicine with high medicinal and economic value. It is mainly used to treat cardiovascular and cerebrovascular diseases [1]. The Chinese pharmacopeia stipulates that the medicinal part of *S. miltiorrhiza* is its dried root. There are two major bioactive components of *S. miltiorrhiza*, lipophilic tanshinones and hydrophilic phenolic acids [2]. More than 40 tanshinones and 20 hydrophilic phenolic acids have been isolated and identified from *S. miltiorrhiza* [3]. The tanshinones, including dihydrotanshinone I (DT-I), cryptotanshinone (CT), tanshinone I (T-I) and tanshinone IIA (T-IIA), are biosynthesized via the mevalonic acid (MVA) and 2-C-methyl-D-erythritol-4-phosphate (MEP) pathways [4, 5]. The phenolic acids, including salvianolic acid B (Sal B) and rosmarinic acid (RA), are biosynthesized through the phenylpropanoid and tyrosine-derived pathways [6, 7]. Most of the key biosynthetic enzyme genes of those pathways have been identified [8, 9]. But the limited supply of bioactive compounds is not able to meet the ever-increasing market demand. Due to this, methods for improving the secondary metabolites biosynthesis have been tried, such as adversity stress, addition elicitor, overexpressing or suppressing genes codifying to enzymes or transcription factors involved in the biosynthetic pathways of these secondary metabolites. Nonetheless, the regulation of gibberellin on secondary metabolites biosynthesis remains unknown.

GAs are growth-promoting phytohormones that regulate numerous growth and developmental processes throughout the whole life cycle of plant, including seed germination, root and stem elongation, and flower development [10]. Since the 1950s, more than 130 GAs have been identified in various plants (http://www.plant-hormones.info/gibberellin_nomenclature.htm) [11]. However, only a few of them, such as G1, G3, G4 and G7, are bioactive [10]. GAs biosynthesis and catabolism pathways in plants have been well characterized. GAs are biosynthesized from the common precursor *trans*-geranylgeranyl diphosphate (GGPP), formed via the MEP pathway. Then, GGPP is modified through the sequential action of two terpene cyclases CPS and KS, followed by oxidation by cytochrome P450 monooxygenases and 2-oxoglutarate-dependent dioxygenases, finally forming GA [12]. Subsequently, GA become functional on plants through its signaling pathways [13]. Binding of GA to the receptor, GID1, causes a conformational change in the N-terminal of protein, which promotes its association with the GRAS domain of DELLA protein. This stable complex enables an efficient SCF^SLY1^ recognition and subsequent degradation of DELLA by the proteasome [14].

The plant-specific GAI-RGA-SCR (GRAS) proteins family function as transcriptional regulators, playing key roles in the GA signaling. Most GRAS proteins contain an N-terminal less-conserved variable region and a C-terminal conserved GRAS domain. Typical GRAS domains comprise 5 conserved sequence motifs: leucine heptad repeats I (LHRI), VHIID, leucine heptad repeats II (LHRII), PFYRE and SAW [15]. Flanked by two leucine-rich regions, the VHIID motif is present in all GRAS family members. Based on their amino acid sequences, the GRAS family is divided into 10 distinct subfamilies: DELLA, SCL3, LAS, SCL4/7, SCR, SHR, SCL9 (LISCL), HAM, PAT1, and DLT [16]. Protein sequences in different subfamily have different characteristics and perform different functions. For example, DELLA proteins possess a conserved DELLA sequence motif in the N-terminal region, which function as GA repressors, acting as key regulatory targets in the GA signaling pathway during the growth regulation [17, 18]. SCL3, in turn, functions as a repressor of DELLA, which can positively regulate the GA signaling pathway and control GA homeostasis during the *Arabidopsis thaliana* root development [19, 20]. The SCL subfamily participates in root cell elongation, also in GA/DELLA signaling and on the stress response mechanisms [15]. The VHIID and PFYRE motifs in the GRAS domain of SHR are essential for the interaction between SCR and SHR [16]. They could form a complex in order to participate in regulating root-related developmental processes in *Arabidopsis* [21, 22]. The PAT1 subfamily has been shown to mediate phytochrome and defense signaling pathways [23]. Moreover, LISCL has two conserved subfamily-restricted acidic motifs in the N-domain and has been reported to be involved in stress response as well as in adventitious root formation in response to auxin [16].

Although GA plays an important role in many aspects of plant growth and development, little is known about its role in regulating secondary metabolism. As diterpenoids, GA and tanshinones have common precursor GGPP [9]. There might be some correlation between the metabolic processes of GA and tanshinones. In addition, GRAS has crucial roles in the GA signaling pathway. Our previous research has shown that 3 *SmGRAS* genes significantly promote tanshinones biosynthesis, also inhibiting GA biosynthesis in hairy roots of *S. miltiorrhiza* [24, 25]*.* Therefore, we speculated that SmGRASs might mediate the regulation of secondary metabolism by the GA signaling in *S. miltiorrhiza*. In order to fully understand the role of the *SmGRASs* family genes on the regulation of secondary metabolism by GA signaling, the hairy roots of wild type *S. miltiorrhiza* were treated with GA, followed with determination of changes in root biomass, root diameter, the contents of tanshinones and phenolic acids. Meanwhile, we also measured the transcriptomic changes and specifically analyzed the transcriptional level alterations of the secondary metabolic pathway and GA signaling pathway genes. Finally, the bioinformatics of all *SmGRAS* family genes in *S. miltiorrhiza* and their responses to GA were analyzed. Our results revealed the possible pathways in which GA regulating secondary metabolism, as well as the response of *SmGRASs* to GA on this secondary metabolism regulation in *S. miltiorrhiza*, providing a reference for the GA signaling pathway to regulate secondary metabolism.

## Results

### GA treatment affects root growth and secondary metabolism

**Fig. 1:**
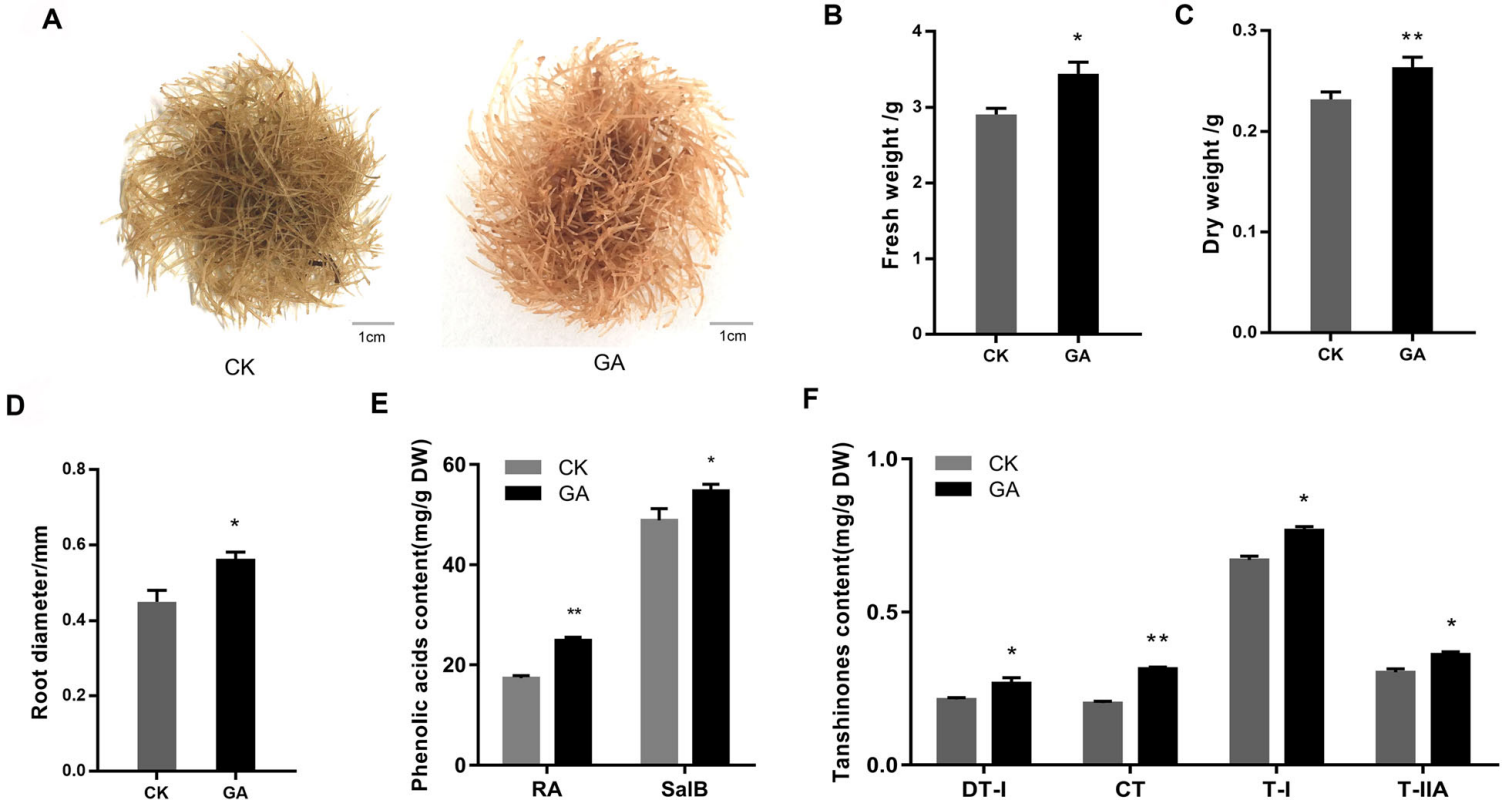
Phenotype, root biomass, root diameter and secondary metabolites contents in *S. miltiorrhiza* hairy roots under CK and GA treatment. **a** Phenotype of hairy roots under CK and GA treatment for 6 days. **b** Fresh weight of hairy roots under CK and GA treatment for 6 days. **c** Dry weight of hairy roots under CK and GA treatment for 6 days. **d** Root diameter of hairy roots under CK and GA treatment for 6 days. **e** Phenolic acids content of hairy roots under CK and GA treatment for 6 days. **f** Tanshinones content of hairy roots under CK and GA treatment for 6 days

The *S. miltiorrhiza* hairy roots were treated with GA during their growth. After 6-day cultivation, the fresh and dry weights of the GA-treated hairy roots were all significantly higher than the controls (Fig. 1a-c), increasing by 19 and 13%, respectively. Moreover, the root diameter of GA-treated hairy roots was significantly higher than that of the controls, increasing by 25% (Fig. 1d). These results indicated that the GA application promoted the growth of hairy roots. In order to investigate the changes of secondary metabolites affected by GA, the contents of phenolic acids and tanshinones were determined after GA treatment. The contents of two phenolic acids and four tanshinones in the hairy roots were all significantly increased in the GA-treated hairy roots (Fig. 1e, f). The increase rates in RA and Sal B were 46 and 14%, respectively, while the increase rates in four tanshinones were 25% (DT-I), 55% (CT), 15% (T-I) and 20% (T-IIA). Collectively, the data indicated that GA treatment promoted the root growth, also increased the accumulation of phenolic acids and tanshinones in the *S. miltiorrhiza* hairy roots.

### Transcriptome-scale analysis of GA-responsive genes

**Fig. 2:**
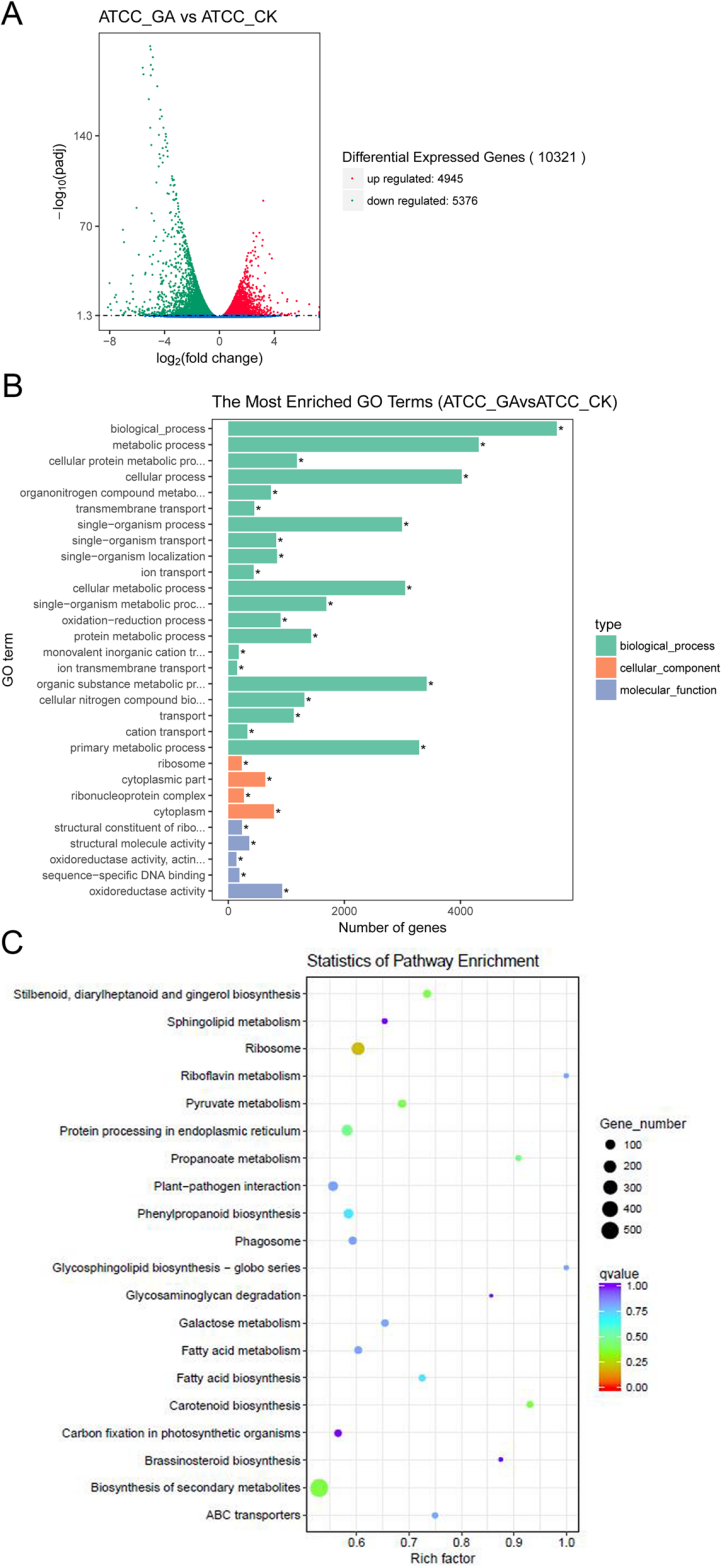
Transcriptomic profiling analysis under CK and GA treatment in hairy roots. **a** Volcano plots of the differentially expressed genes (DEGs) in the comparison of GA (ATCC-GA) and CK (ATCC-CK) hairy roots. **b** Functional gene ontology (GO) term classifications of DEGs from comparisons of GA and CK hairy roots. **c** Kyoto Encyclopedia of Genes and Genomes (KEGG) classification of DEGs in the comparisons of GA and CK hairy roots

In order to gain a comprehensive overview of the GA-responsive genes, we performed a transcriptomic analysis of CK and GA-treated hairy roots. 10321 differentially expressed genes (DEGs) were found and annotated in the volcano plot. The comparison of CK and GA-treated hairy roots revealed that 4945 genes were GA-induced, and 5376 were GA-repressed (Fig. 2a). To verify the results reliability from RNA-seq, 10 genes were randomly selected for Quantitative Reverse-Transcription PCR (qRT-PCR) analysis, whose results were consistent with the RNA-seq results, indicating that these RNA-seq data was reliable (Fig. S1, 2 and 3, Table S1, 2). The global functional analysis of the DEGs revealed that the “biological processes”, “metabolic processes” and “cellular processes” were the top three categories in the most enriched gene ontology (GO) terms (Fig. 2b, Table S3). Additionally, these DEGs identified were further assessed using the Kyoto Encyclopedia of Genes and Genomes (KEGG). The most significantly enriched term was “biosynthesis of secondary metabolites”, followed by “ribosome”, “plant-pathogen interaction” and some “primary and secondary metabolic pathways” (Fig. 2c, Table S4).

### Secondary metabolism pathway genes in response to GA treatment

**Fig. 3:**
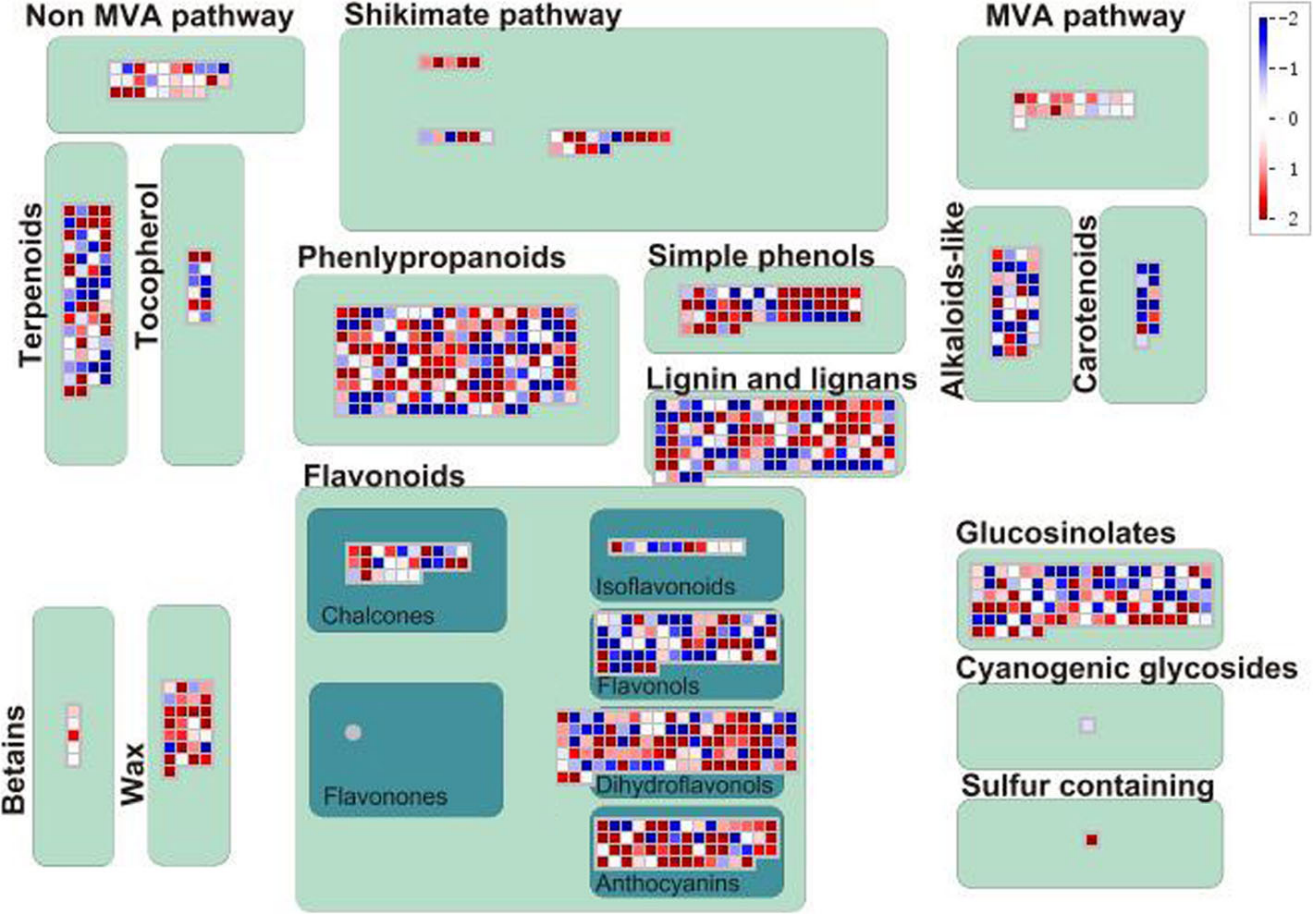
DEGs of secondary metabolism pathway between CK and GA treatment hairy roots. DEGs marked in red indicated they were GA-induced, while the blue ones were GA-repressed

In order to further explore the effect of GA on secondary metabolism, we used the Mapman program to analyze the transcriptomic data (Fig. 3). The results showed that most DEGs of secondary metabolism were GA-induced, especially the shikimate pathway, MVA pathway, simple phenols, betaines, wax, and anthocyanins. Diterpenoids, such as GA and tanshinones, are biosynthesized by MVA and MEP pathways. Most of the DEGs in the MVA pathway were GA-induced at the transcriptional level, which might be related to the fact that GA-induced tanshinones biosynthesis. On the other hand, phenolic acids are mainly biosynthesized by shikimate and phenylpropane pathways. The results showed that most of the shikimate pathway DEGs and some of the phenylpropane pathway DEGs were induced by GA. In addition, GA also affected the biosynthesis pathways of flavonoids and alkaloids-like, wax and glucosinolates. In summary, GA regulated the gene transcription in many secondary metabolites’ biosynthetic pathway.

### GA biosynthetic and signaling pathways genes in response to GA treatment

**Fig. 4:**
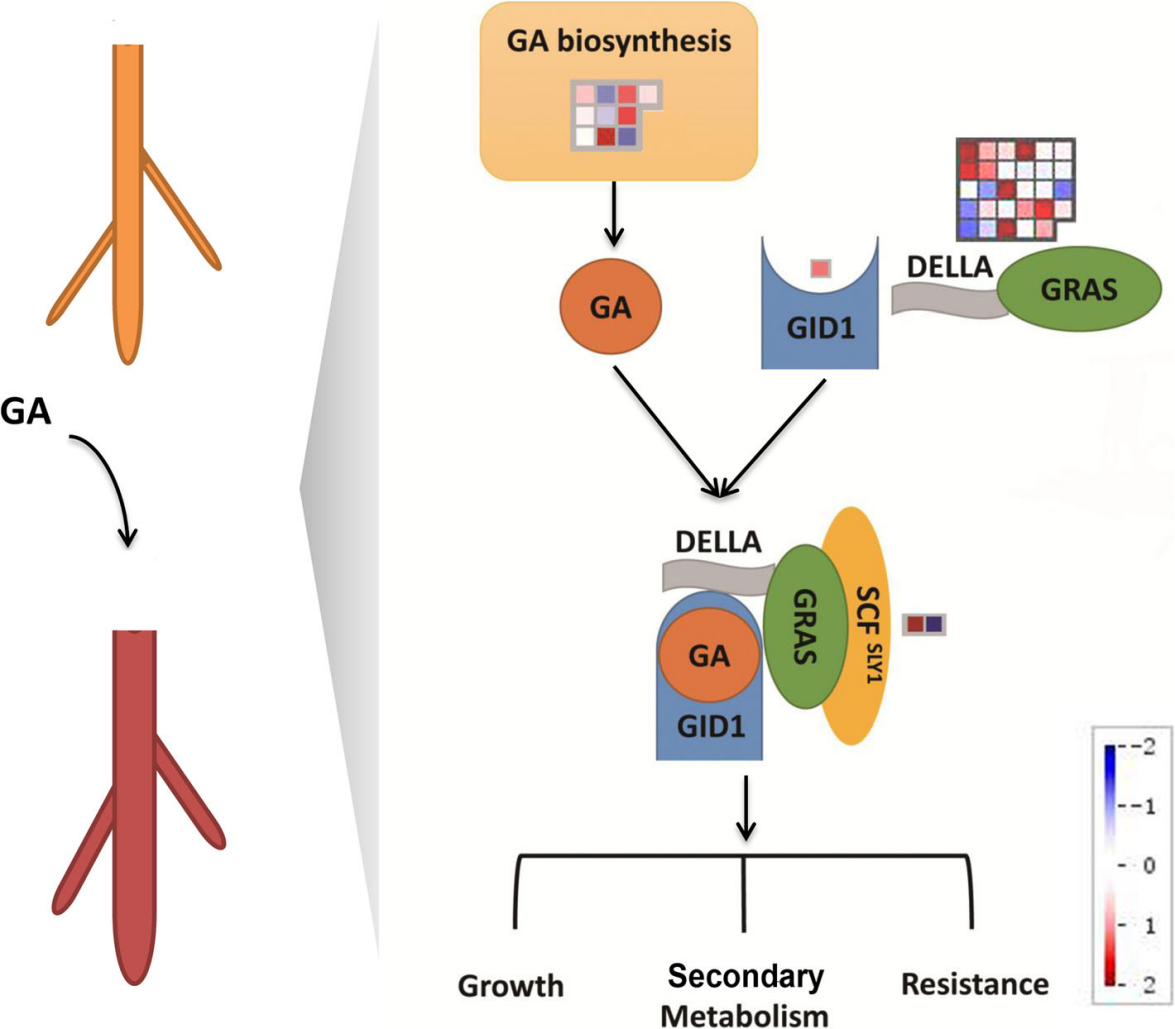
A model for a possible mechanism of the regulation of GA to root growth and secondary metabolism. DEGs marked in red indicated they were GA-induced, while the blue ones were GA-repressed

GA treatment can directly affect the regulation of the GA signaling pathway in plant. To investigate the effect of this treatment on GA biosynthesis and signaling pathway, we further analyzed and summarized the DEGs involved in GA biosynthetic and signaling pathway (Fig. 4). The results showed that the transcriptional changes in GA biosynthetic pathway genes were diverse, some DEGs had increased their expression levels, whereas others had it decreased. In the downstream GA signaling pathway, the expression of GID1 (GA receptor) was increased. Coherently, the expression levels of most GRAS family genes, which were the key regulators of the GA signaling pathway, were increased as well. Nevertheless, the expressions of F-box proteins SCF in the GA signaling pathway were different under GA treatment. In conclusion, GA could effectively regulate the expressions of biosynthetic pathway genes and downstream signaling pathway genes, especially the *SmGRAS* family genes, and further regulating many downstream physiological processes, such as cell growth, secondary metabolism, and plant resistance.

### Identification and phylogenetic analysis of GRAS proteins in *S. miltiorrhiza*

**Fig. 5:**
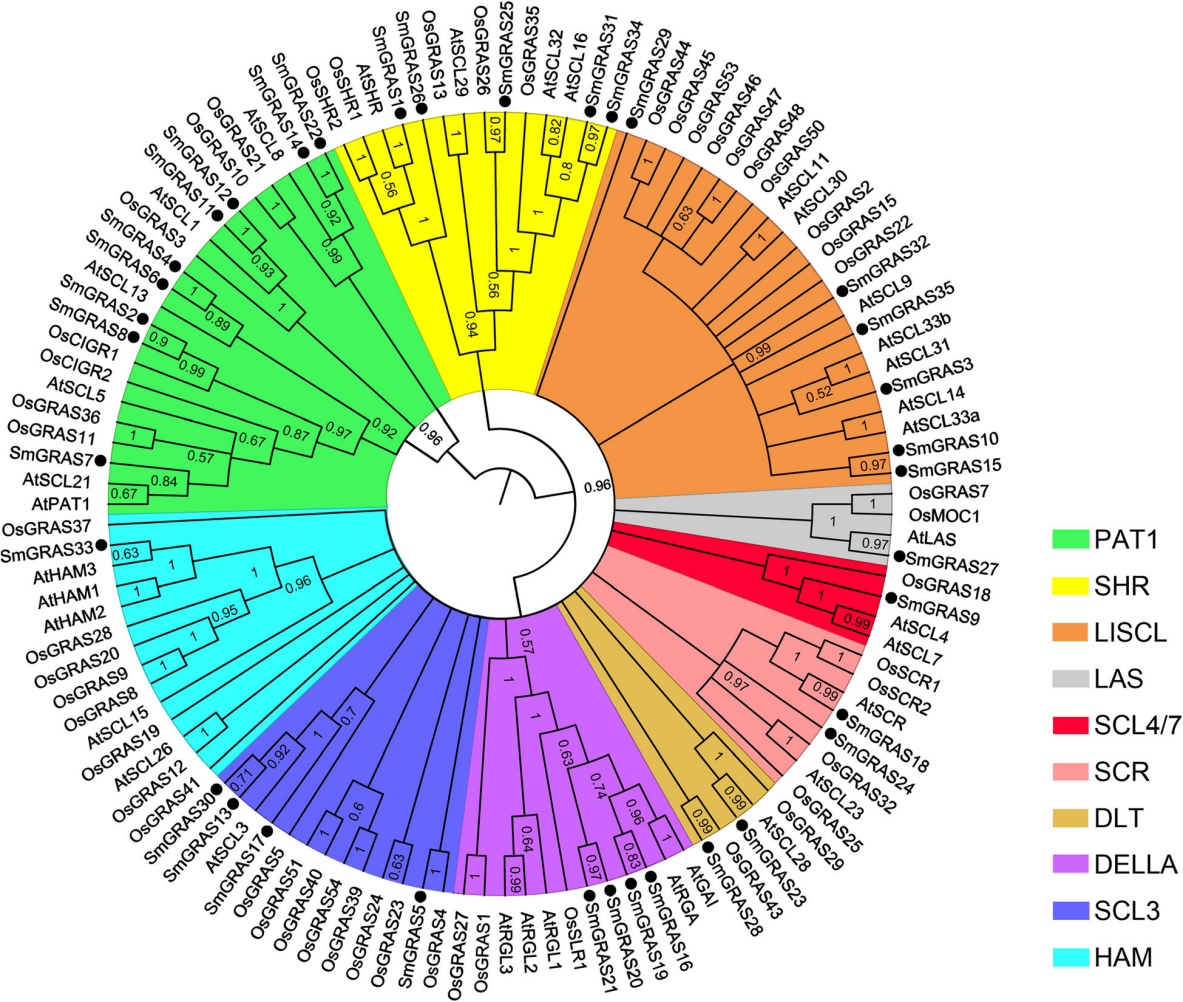
The phylogenic tree of GRAS transcription factors used the Neighbor-Joining (NJ) method of *S. miltiorrhiza*, *Arabidopsis*, and rice. Different subfamilies were marked with different background colors

In order to study the roles of SmGRAS in GA regulation of root growth and secondary metabolism of *S. miltiorrhiza*, we conducted a comprehensive analysis of the *SmGRAS* family genes in this species. We used HMMER to screen the protein sequences based on the HMM profiles from the *S. miltiorrhiza* genome database to identify putative GRAS proteins. Thirty-five SmGRAS proteins were identified from it, which were named GRAS1 ~ 35. The putative amino acid sequences of *SmGRAS*1 ~ 35 contained the conserved GRAS domain for the GRAS protein [15]. To study the evolutionary relationships of *SmGRAS* genes, phylogenetic tree analysis with dicotyledons of *Arabidopsis* and monocotyledons of rice was constructed, which revealed that the SmGRAS proteins were divided into 10 subfamilies (Fig. 5). Among these GRAS family proteins, there were 9 proteins from the PAT1 subfamily, 6 proteins from the LISCL subfamily, 5 proteins from the SHR subfamily, 4 proteins from the SCL and DELLA subfamilies, 2 proteins from the DLT and SCR subfamilies, bedides the other 3 subfamilies only having 1 protein. Therefore, we speculated that these *SmGRAS* genes might be similar to *Arabidopsis GRAS* genes of the same subfamily. They might be involved in the GA signaling pathway, root and stem cortex development, light morphogenesis and stress tolerance.

### SmGRAS proteins sequence alignments and conserved motifs

**Fig. 6:**
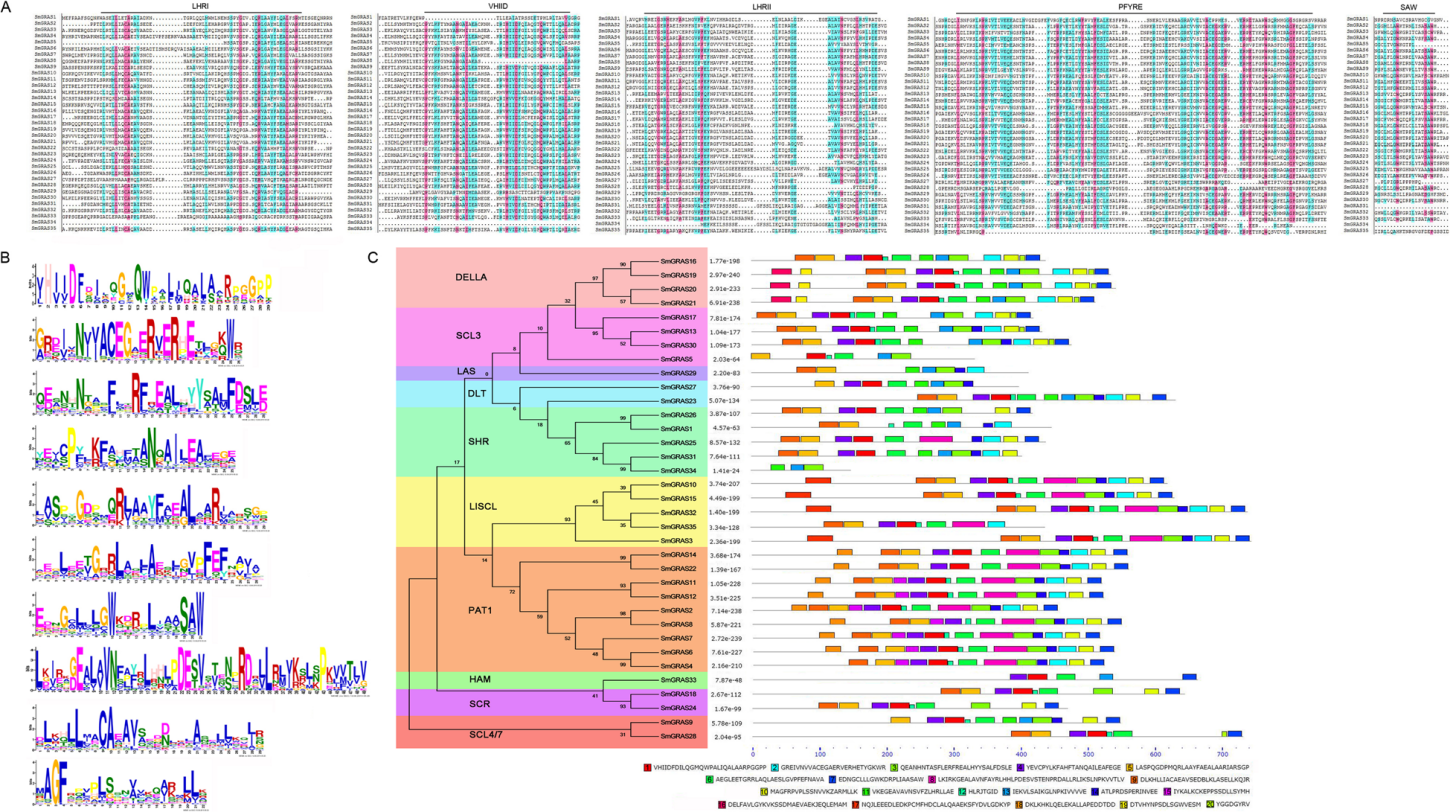
Conserved motifs and gene structures of *SmGRAS* transcription factors in *S. miltiorrhiza.* **a** Multiple sequence alignment of the GRAS domain from SmGRAS proteins. **b** Subfamily-specific motifs of SmGRAS. **c** Gene structures of *SmGRAS*

In order to further confirm that these 35 genes belong to the GRAS family, we used DNAMAN and online MEME to perform multiple sequences alignment and conservative domain analysis on them. The multiple sequences alignment result showed that the amino acid sequences of all these 35 proteins have high identities (Fig. 6a). Almost all those SmGRAS proteins contain conserved GRAS domain, as LHRI, VHIID, LHRII, PFYRE and SAW. We also used online MEME to identify the conserved motifs of full-length SmGRAS proteins (Fig. 6b, c) and 10 most conserved motifs located in the GRAS domain were shown in Fig. 6b. In Fig. 6c, there are 20 conserved motifs were identified in the 35 SmGRAS proteins. The VHIID and SAW motifs which were the most conserved motifs in the GRAS domain, were found in all SmGRAS proteins. The LHRI, LHRII, and PFYRE motifs were found in most of SmGRAS proteins, while other 15 motifs were found just in some SmGRAS proteins. The results suggested that all these 35 SmGRAS proteins have the conserved GRAS domain, however, SmGRAS proteins from different subfamilies have different motifs, probably in order to perform different functions.

### Structural analysis of *SmGRAS* genes

To further analyze the structural components and physicochemical properties of 35 *SmGRAS* genes, we conducted an in-depth analysis with ExPASy (Table 1). The result showed that the open reading frame length of most *SmGRAS* family genes was over 1000 bp. About the 35 *SmGRAS* genes, *SmGRAS34* was the shortest one, with 453 bp, while the longest one was the *SmGRAS3*, with 2247 bp. The protein length ranged from 150 (*SmGRAS34*) to 748 amino acids (*SmGRAS3*), and the molecular weight ranged from 16.8 to 84.0 kDa. The protein isoelectric points ranged from 4.8 (*SmGRAS20*) to 9.0 (*SmGRAS35*), with most of them ranging from 5 to 6. The majority of *SmGRAS* proteins contained just 1 exon, while some contained 2 exons. Only the *SmGRAS2*8 has 4 exons and *SmGRAS35* has 5 exons.

**Table 1:** The information of 35 SmGRAS transcription factors identified in *S.miltiorrhiza* genome

Gene	Gene ID	ORF length (bp)	Protein length (aa)	Mw (Da)	pI	Exon no.
SmGRAS1	SMil_00008598	1470	489	54680.21	5.813	1
SmGRAS2	SMil_00025700	1380	459	51215.14	6.184	1
SmGRAS3	SMil_00006393	2247	748	83952.38	4.953	1
SmGRAS4	SMil_00023920	1581	526	58112.72	5.427	1
SmGRAS5	SMil_00019115	1041	335	37542.27	5.774	1
SmGRAS6	SMil_00022108	1626	541	59758.38	5.462	1
SmGRAS7	SMil_00020530	1563	520	57762.17	6.031	1
SmGRAS8	SMil_00027641	1662	553	62006.62	5.661	2
SmGRAS9	SMil_00022663	1653	550	60292.19	5.429	2
SmGRAS10	SMil_00013932	1872	623	70517.82	6.435	1
SmGRAS11	SMil_00010266	1575	524	58338.81	5.321	1
SmGRAS12	SMil_00005963	1584	527	58587.25	5.121	1
SmGRAS13	SMil_00014572	1308	435	48756.82	6.053	1
SmGRAS14	SMil_00014379	1692	563	61920.17	6.916	1
SmGRAS15	SMil_00013931	1905	634	71762.76	6.203	1
SmGRAS16	SMil_00019995	1326	441	48523.73	5.853	1
SmGRAS17	SMil_00012335	1269	422	47557.06	6.496	1
SmGRAS18	SMil_00023609	1944	647	70502.62	5.986	2
SmGRAS19	SMil_00027306	1617	538	59384.69	5.694	1
SmGRAS20	SMil_00025750	1628	545	59449.16	4.832	1
SmGRAS21	SMil_00015621	1548	515	56501.40	5.806	1
SmGRAS22	SMil_00016132	1695	564	61825.58	5.768	2
SmGRAS23	SMil_00019107	1911	636	70786.90	6.863	1
SmGRAS24	SMil_00011905	1419	472	52047.14	6.533	1
SmGRAS25	SMil_00024909	1326	441	48314.18	5.097	1
SmGRAS26	SMil_00017304	1266	421	47024.52	5.397	1
SmGRAS27	SMil_00018880	1206	401	44337.81	8.832	2
SmGRAS28	SMil_00018052	2205	734	81680.17	6.038	4
SmGRAS29	SMil_00012105	1248	415	44237.29	9.695	2
SmGRAS30	SMil_00000751	1440	479	53599.74	6.116	1
SmGRAS31	SMil_00012472	1218	405	44533.13	5.761	1
SmGRAS32	SMil_00017280	2235	744	83995.86	5.793	1
SmGRAS33	SMil_00000430	1998	665	71197.52	5.928	2
SmGRAS34	SMil_00006319	453	150	16797.00	4.990	1
SmGRAS35	SMil_00004080	1326	441	49830.65	9.001	5

### Expression analysis of *SmGRAS* genes in response to GA treatment

**Fig. 7:**
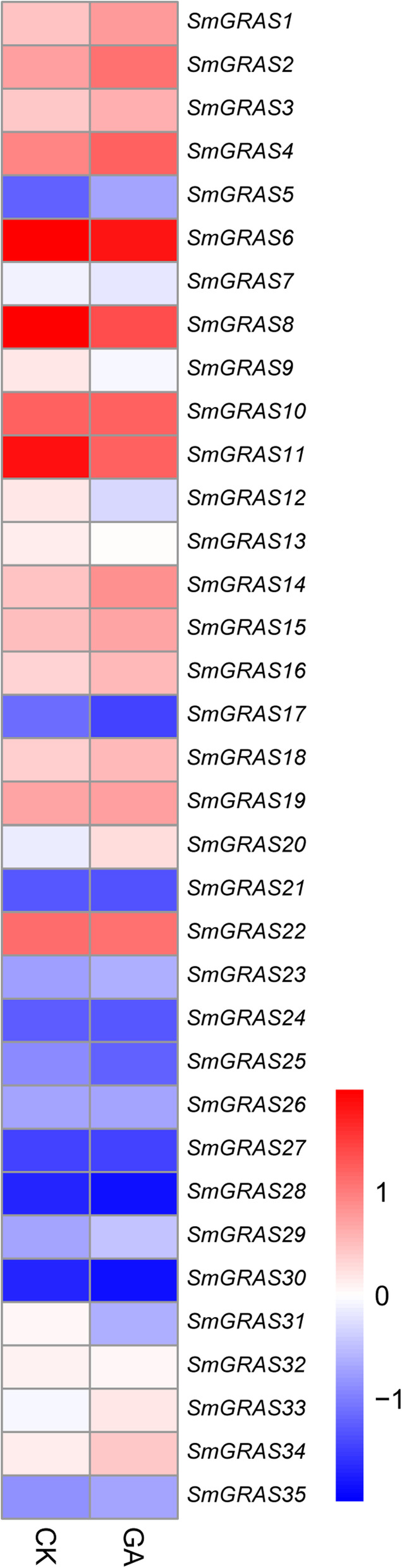
Gene expression profiles of *SmGRAS* members under control and GA treatment. Genes marked in red indicated they were GA-induced, while the blue ones were GA-repressed

As the key regulator of the GA signaling pathway, the transcription levels of *GRASs* were significantly affected by GA. We comprehensively analyzed the transcription level changes of these 35 *SmGRAS* family genes after 100 μm GA treatment for 2 h. So these expression levels changed a lot. The heatmap showed that 15 *SmGRAS* genes were GA-induced, while 11 *SmGRAS* genes were GA-repressed, besides other 9 genes did not change significantly under GA treatment (Fig. 7). The most significantly increased of these was *SmGRAS5*, which increased 4-fold expression levels, followed by *SmGRAS20* (2.3-fold) and *SmGRAS14* (2.1-fold). At the other hand, the most significantly reduced in those genes was the *SmGRAS28*, which reduced about 90% in comparison with the control. The expressions of *SmGRAS31*, *SmGRAS8*, *SmGRAS11*, and *SmGRAS12* were all fell by more than half.

## Discussion

It is well known that bioactive GAs are diterpene phytohormones that regulate plant growth and development throughout the whole life cycle [14]. There are many reports concerning the important roles of GA in plant growth, development and stress tolerance [10–13], but just few about the relationship between GA and secondary metabolism. In addition, GA shares the identical biosynthetic pathway and precursor substances with diterpenoid metabolites tanshinones, which were the main secondary metabolites of *S. miltiorrhiza* [9]. We speculated that there might be some correlation between GA and tanshinones. Therefore, we treated the hairy roots of *S. miltiorrhiza* with GA and found that it not only promoted the root growth but also increased the accumulation of tanshinones and phenolic acids. These results make us beginning to pay attention to the specific mechanism of GA regulating secondary metabolism.

It is also known that GA regulates many growth and development processes such as seed germination, root and shoot elongation, metabolism, stress tolerance, flowering and fruit patterning [10, 12]. The GA signaling pathway includes the biosynthesis of the active GA, perception, signal transduction and inactivation [12]. It is now clear that GAs accumulate in the elongating endodermal cells of *Arabidopsis* root, playing central roles in growth regulation through the key transcription factor DELLA (GRAS family member) [10, 13]. Several genes of the GRAS transcription factor family in the GA signaling pathway have been reported to regulate the development of the cortex in the root. For instance, GRAS family genes form a SHR-SCR-SCL3 complex, which regulates the middle cortex formation in *Arabidopsis* root [26]. SCL28 also plays an important role in root growth response to stress-induced microtubule organization in *Arabidopsis* [27]. In addition, the GA-mediated growth control and energy metabolism have an interaction, which coordinates cell wall extension, secondary metabolism and lipid metabolism in *Arabidopsis* [28]. Similarly, the overexpression of *HaGRASL* reduces the GAs metabolic flow in *Arabidopsis*, so that modification could be relevant in axillary meristem development [29].

Based on this reasoning, after treating wild-type hairy roots of *S. miltiorrhiza* with GA, we found that it promoted the root growth, enhanced the accumulation of tanshinones and phenolic acids. In order to explore the possible molecular mechanism, we performed transcriptomic analysis, whose results showed that the gene response of the secondary metabolic pathway was significant after GA treatment. So we speculated that GA regulated the accumulation of secondary metabolites by regulating the expression of genes involved in the secondary metabolites biosynthetic pathway. After further analysis of the DEGs in secondary metabolic pathways, we found that most DEGs in MVA, shikimate and phenylpropane pathways are GA-induced at the transcriptional level. This result also explains a series of composition content changes in the hairy root of *S. miltiorrhiza* at the transcriptional level after GA treatment, such as the increased tanshinones and phenolic acids contents. In addition, since our previous research found that 3 *SmGRAS* genes can promote the biosynthesis of tanshinones and inhibit the biosynthesis of GA, we hypothesize that GRAS core regulators of the GA signaling pathway would be involved in the regulation of GA on the secondary metabolism [24, 25]. Therefore, we also deeply analyzed the expressions of DEGs in the GA signaling pathway. The results showed that the gene expression levels of most *GRAS* family genes and GA receptor *GID1* in the pathway were significantly increased. Therefore, it is speculated that GA induction affects the GA signaling pathway, also causing the response of *SmGRASs* in the GA signaling pathway, regulating some downstream physiological processes such as growth, secondary metabolism and stress response.

In order to study the roles of GRAS during the GA regulation on plant growth and secondary metabolism, we used HMMER to search for all SmGRAS protein sequences based on the HMM profiles from the *S. miltiorrhiza* genome database. *SmGRAS* was found to be a large family of transcription factors with 35 genes. These SmGRAS proteins were systematically analyzed in relation to the GRAS family proteins of monocotyledons rice and dicotyledons *Arabidopsis*. The phylogenetic tree revealed that these SmGRASs had gathered into 10 subfamilies, PAT1 (9 SmGRASs), LISCL (6), SHR (5), DELLA (4), SCL3 (4), SCR (2), DLT (2), HAM (1), SCL4/7 (1) and LAS (1). Orthologous genes generally retained similar functions. The PAT1 subfamily has been reported to mediate phytochrome, light and defense signaling pathways [15]. The LISCL subfamily was involved in stress response and response to auxin. SHR, SCR and SCL3 formed a complex to regulate middle cortex formation. DELLA has a central role in suppressing GA signaling [30]. DLT was involved in the brassinosteroid signaling, SCL4/7 was involved in the response to environmental stresses and LAS participated in the formation of lateral shoots [15]. Therefore, we speculated that SmGRASs might also be involved in the regulation of the above processes in *S. miltiorrhiza*. In addition, we further analyzed the conservative sequence. Most SmGRAS proteins are clustered into similar subfamilies, they share similar motifs, and also have family-specific functions. The conservative sequence is slightly different in different subfamilies. For example, motif 15 was only present in subfamily PAT1; motifs 10 and 16 were only present in DELLA subfamily; motif 17 was only found in subfamily LISCI, indicating their specific functions. The distribution of conserved motifs reflects the relations between different subfamilies. For example, motifs 13 and 20 were found, while motifs 8, 15 and 17 were completely lost in subfamilies SCL3 and DELLA. These results indicated the evolutionary relationship between subfamilies SCL3 and DELLA is very close. Therefore, the structural analysis also provided a clue to determine the sub-family of *GRAS* genes and its ancient origin.

Finally, we analyzed the responses of all *SmGRAS* family genes to GA induction. The expression analysis indicated that most *SmGRAS* genes were significantly induced or inhibited in GA induction. In *Arabidopsis*, DELLA proteins act as repressors of GA-responsive plant growth and three *DELLA* genes (*SmGRAS16*, *19* and *20*) were induced by GA treatment. SCL3, as the repressor of DELLA, could positively regulate the GA signaling pathway and also control GA homeostasis in *Arabidopsis* root [20]. *SmGRAS13*, *17* and *30* are orthologous to *SCL3* gene in *S. miltiorrhiza*, so that it was evidently downregulated under GA treatment. Therefore, we speculated that *SmGRAS* proteins of different subfamilies played different regulatory roles through the GA signaling pathway, regulating many physiological processes in *S. miltiorrh*iza. Finally, we also proposed a model diagram for the GA signaling pathway regulation. The results showed that GA could regulate the expressions of biosynthetic pathway genes as well as the downstream signaling pathway genes, mainly *SmGRAS* transcription factors, and further regulating many downstream physiological processes, such as cell growth, secondary metabolism, and plant resistance.

## Conclusions

We found that GA could promote the growth of *S. miltiorrhiza* hairy roots, also increasing the accumulation of tanshinones and phenolic acids. Many DEGs in metabolic processes, especially the secondary metabolic processes, as well as the GA signaling pathway regulator *SmGRASs* could significantly be induced under GA treatment. The key regulatory factors SmGRASs play important roles on the GA-regulated root growth and secondary metabolism in *S. miltiorrhiza*. Although providing a range of references for understanding the molecular mechanism that exists behind the GA regulating secondary metabolism, further studies are needed for a better acquaintance of this complex mechanism.

## Methods

### Plant materials and treatment

The *S. miltiorrhiza* leaves were took from the sterile seedling line preserved at our laboratory at Northwest A&F University in Yangling, Shaanxi Province, China. The establishment of *S. miltiorrhiza* hairy roots was derived from aseptic leaves of *S. miltiorrhiza* infected with *Agrobacterium rhizogenes* (ATCC15834), as previously reported [31]. Samples of the hairy roots (0.3 g fresh weight) were cultured in 100 mL beaker flasks contains 50 mL of the 6,7-V liquid medium on an orbital shaker 120 rpm·min^− 1^, 25 °C in the dark, sub-cultured every 30 days.

For GA treatment, GA_3_ (Sigma, CA, USA) stock solution was filter sterilized through 0.22 μm filters and added to cultures of 21-day-old hairy roots, to a desired final concentration of 100 μM. After 2 h and 6 days of GA treatment, hairy roots were collected for the qRT-PCR and HPLC analysis. Hairy roots without GA treatment were used as control samples. All treatments were performed in three independent biological replicates.

### HPLC analysis

The 21-day-old hairy roots were treated with 100 μM GA_3_ for 6 days and collected in three biological replicates. Then 0.04 g of dried hairy roots was extracted and analyzed by HPLC, according to the general method in our laboratory, which was previously described [32]. The GA_3_ concentration analysis was measured by HPLC as previously described as well [33].

### RNA-seq library construction and sequencing

On 2 h after GA treatment, the GA-treated hairy roots (GA) and control samples (CK) were collected from three biological replicates and analyzed by transcriptomic-based technology. Total RNA was extracted using the RNA extraction kit following the manufacturer’s instruction (Tiangen Biotech, Beijing, China). After measuring the quality, strand-specific RNA-Seq libraries were constructedand sequenced on the Illumina PE150 platform (Novogene, Tianjin, China). The high-quality clean data were calculated and used for downstream analysis.

### Identification of DEGs and functional enrichment

The reference genome and gene model annotation files of *S. miltiorrhiza* were downloaded from genome websites [34]. Fragments per kilobase of transcript per million mapped reads (FPKM) were used to determine the relative expression levels of each gene. DEGs were determined using the DESeq R package [35]. The expression levels of DEGs were considered significantly differentially expressed genes with an adjusted *P*-value< 0.05 and |fold-change| ≥ 2. The GO term enrichment of DEGs was evaluated using the GOseq R package [36]. Statistical enrichment of DEGs in the KEGG was identified with KOBAS [37]. Then Mapman program was used to analyze the transcriptome data of metabolic and signal pathways.

### qRT-PCR validation

A total of 10 DEGs were randomly chosen to verify the RNA-seq data (Fig. S3). Ten DEGs specific primers were designed by primer 5 (Table S5). qRT-PCR was performed on a real-time PCR system (Bio-Rad CFX96, USA), according to our previous methods [24]. The *SmActin* gene was used as the endogenous control [38]. All assays for each gene were performed in triplicate under identical conditions.

### Identification of GRAS family members in *S. miltiorrhiza* and phylogenetic analysis

A Hidden Markov Model (HMM) of the GRAS domain (PF03514) was downloaded from the Pfam database (http://pfam.xfam.org). HMM algorithm (HMMER) was used (http://www.hmmer.org/) to search for the GRAS domain in the *S. miltiorrhiza* genome database [39], with an E-value<1e^− 6^. Multiple sequence alignments of GRAS protein sequences from *S. miltiorrhiza*, *O. sativa* and *A. thaliana* (https://phytozome.jgi.doe.gov/pz/portal.html) were performed using the Clustal X program. A phylogenetic tree based on the alignment was constructed with MEGA 6.0 by the neighbor-joining method with the bootstrap test (*n* = 500 replications) [40].

### Analysis of conserved motifs and gene structures

Gene Structure Display Server (http://gsds.cbi.pku.edu.cn/) was used to obtain the gene structure of introns and exons based on the CDS and corresponding to genic sequences. GRAS domains were aligned and the conserved sites were checked manually for their corresponding amino acid residues, which were shaded using DNAMAN software. Conserved motif analysis of SmGRASs was performed by online MEME tools with 20 motif numbers. The theoretical isoelectric point (pI) and molecular weight (Mw) were predicted by the ExPASy server (http://web.expasy.org/computepi/).

## Supplementary information

**Additional file 1: Figure S1.** The classification of raw reads.

**Additional file 2: Figure S2.** The pearson correlation between sample replicates.

**Additional file 3: Figure S3.** Validating the different expression levels of the identified genes from RNA-seq data by qRT-PCR.

**Additional file 4: Table S1.** Data output quality list.

**Additional file 5: Table S2.** Reads and reference genome comparison list.

**Additional file 6: Table S3.** GO enrichment of DEGs between CK and GA-treated hairy roots. (XLS 3958 kb)

**Additional file 7: Table S4.** KEGG Pathwayenrichment of DEGs between CK and GA-treated hairy roots. (XLS 246 kb)

**Additional file 8: Table S5.** Primers used for qRT-PCR.

## Data Availability

The data sets supporting the conclusions of this article are included within the article and its additional files. Raw reads data has been deposited in NCBI under accession number PRJNA663993.

## Abbreviations

GRAS: GAI-RGA-SCR
GA: Gibberellic acid
DT-I: Dihydrotanshinone I
CT: Cryptotanshinone
T-I: Tanshinone I
T-IIA: Tanshinone IIA
MVA: Mevalonic acid
MEP: 2-C-methyl-D-erythritol-4-phosphate
SAs: Salvianolic acids
Sal B: Salvianolic acid B
RA: Rosmarinic acid
GGPP: Trans-geranylgeranyl diphosphate
LHR: Leucine heptad repeats
DEGs: Differentially expressed genes
qRT-PCR: Quantitative Reverse-Transcription PCR
GO: Gene ontology
KEGG: Kyoto Encyclopedia of Genes and Genomes
FPKM: Fragments per kilobase of transcript per million mapped reads
HMM: Hidden Markov Model
