# Supplementary figures and images for: Transcriptomic analysis reveals the GRAS family genes respond to gibberellin in Salvia miltiorrhiza hairy roots
Source: BMC Genomics. 2020 Oct 27;21:727. doi: 10.1186/s12864-020-07119-3 (PMC7590604; doi:10.1186/s12864-020-07119-3)

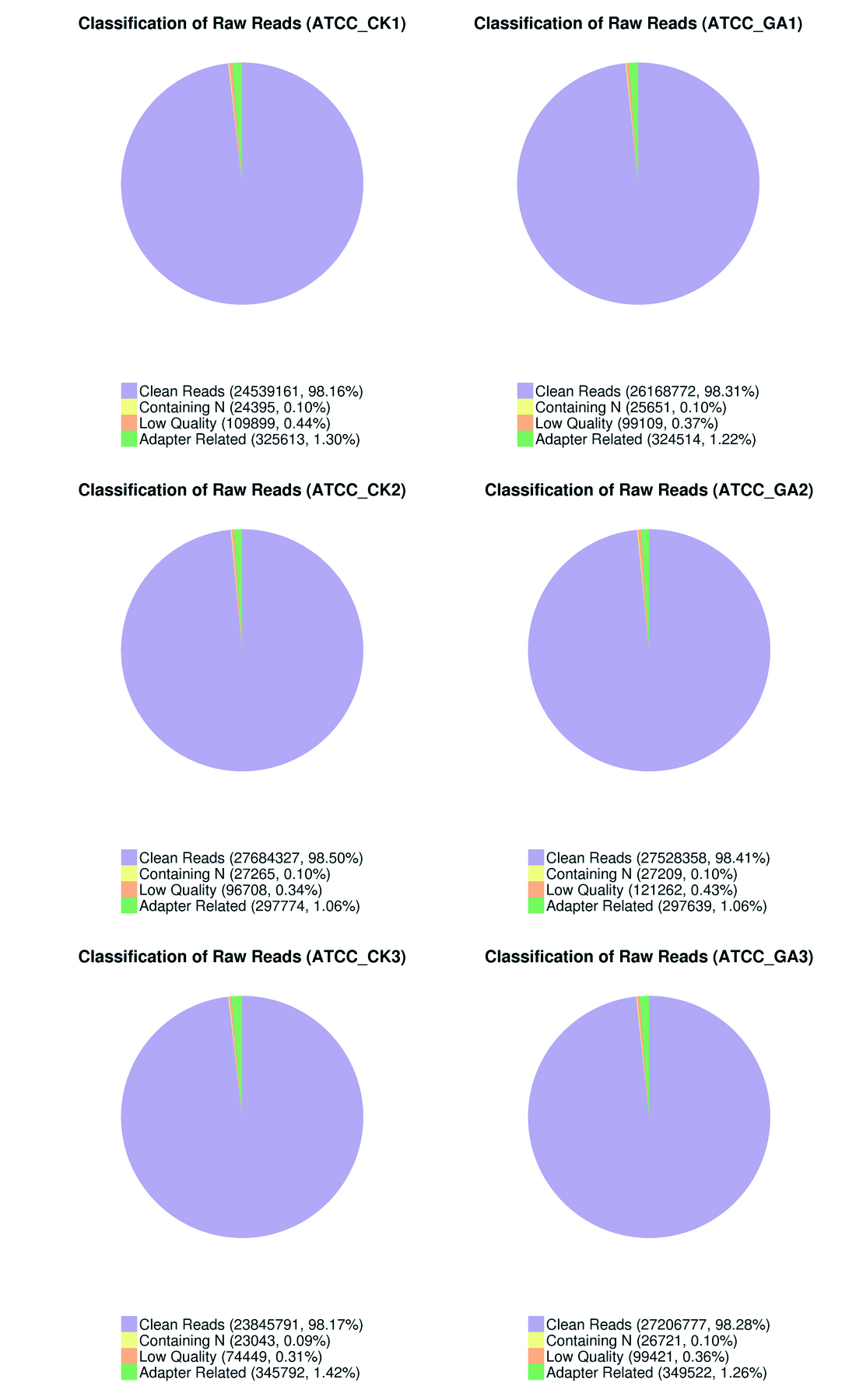

Supplement: Supplementary file 1 — Additional file 1: Figure S1. The classification of raw reads. [file 12864_2020_7119_MOESM1_ESM.tif]

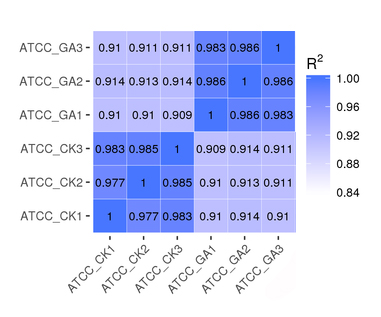

Supplement: Supplementary file 2 — Additional file 2: Figure S2. The pearson correlation between sample replicates. [file 12864_2020_7119_MOESM2_ESM.tif]

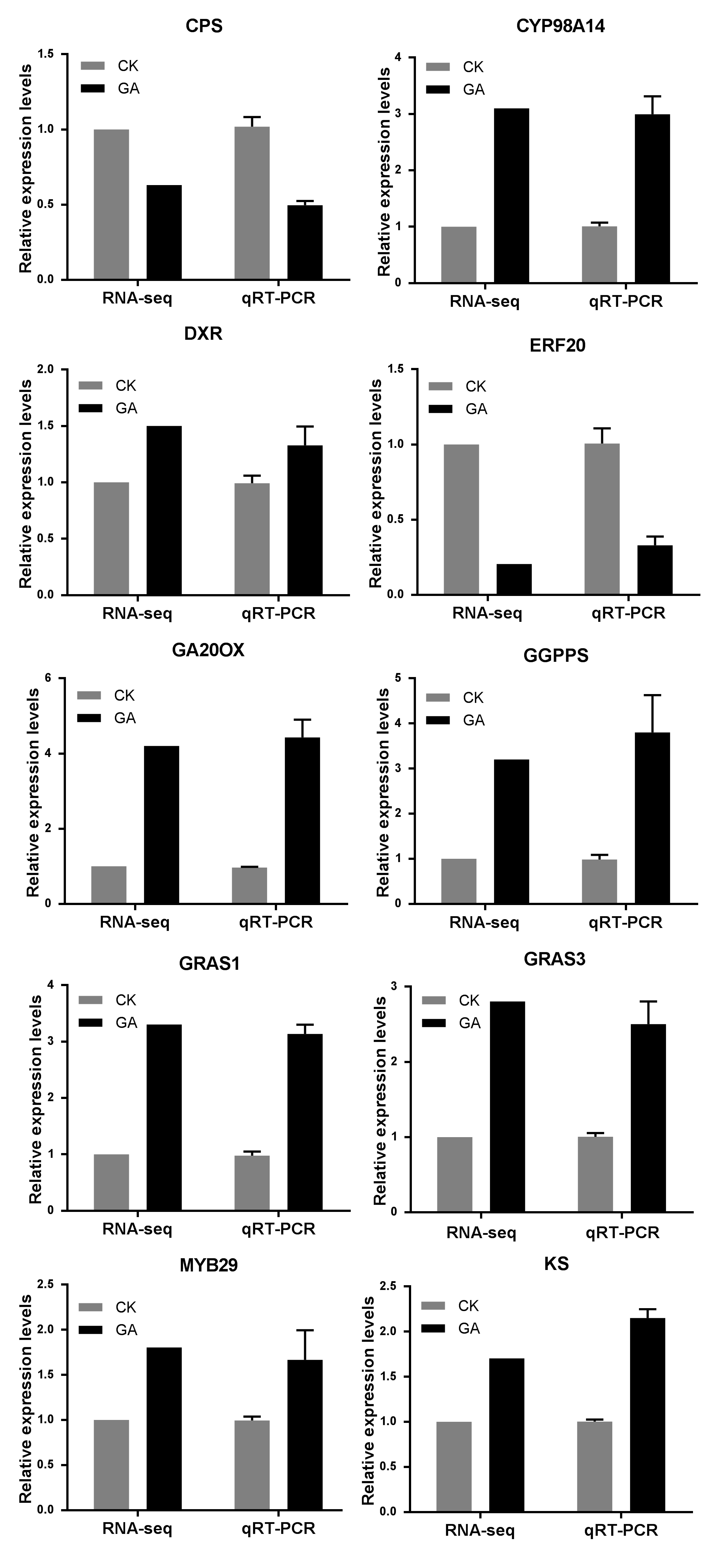

Supplement: Supplementary file 3 — Additional file 3: Figure S3. Validating the different expression levels of the identified genes from RNA-seq data by qRT-PCR. [file 12864_2020_7119_MOESM3_ESM.tif]
