# Supplementary material for: Transcriptomic analysis reveals the GRAS family genes respond to gibberellin in Salvia miltiorrhiza hairy roots
Source: BMC Genomics. 2020 Oct 27;21:727. doi: 10.1186/s12864-020-07119-3 (PMC7590604; doi:10.1186/s12864-020-07119-3)
Supplement: Supplementary file 8 — Additional file 8: Table S5. Primers used for qRT-PCR. [file 12864_2020_7119_MOESM8_ESM.docx]

**Table S5. Primers used for qRT-PCR**

| Gene name | Primer name | Sequence (5’ to 3’) |
| --- | --- | --- |
| SmACT | ACT-F | GGTGCCCTGAGGTCCTGTT |
|  | ACT-R | AGGAACCACCGATCCAGACA |
| SmCPS1 | CPS1-F | CCACATCGCCTTCAGGGAAGAAAT |
|  | CPS1-R | TTTATGCTCGATTTCGCTGCGATCT |
| SmCYP98A14 | CYP98A14-F | CCTCAACGTCGTCGTTTCCA |
|  | CYP98A14-R | AGTCCGCCCAAATCAAATCC |
| SmDXR | DXR-F | CATGCGTTTGCCTATTCTGTAC |
|  | DXR-R | ACTAAGAACTCCGGTCATGGTG |
| SmERF20 | ERF20-F | TCCTCCAACACCGAAGAT |
|  | ERF20-R | TTCTGCAAGGACGAATCTC |
| SmGA20OX1 | GA20OX1-F | AAGGAGGAGAAGAGGAAGG |
|  | GA20OX1-R | GCGTATATGACCGTTGGAT |
| SmGGPPS1 | GGPPS1-F | ACAAGACCACGTATCCCAAGC |
|  | GGPPS1-R | TCTGCCTATGTGCAATGTAATCG |
| SmGRAS1 | GRAS1-F | GCCTACGACCAATCCTCCTACTCCA |
|  | GRAS1-R | CGGTCATGCGGCTGAACAATGC |
| SmGRAS3 | GRAS3-F | CCTTGCTCTACGTGCTGCTGAGAA |
|  | GRAS3-R | ACTGCTGCTTGTATACTCGCTGGA |
| SmMYB89 | MYB89-F | CCGCCACCATTAACTCAA |
|  | MYB89-R | GAGCAGAAGGAAGCAGAG |
| SmKS | SmKS-F | TTAGTTTTGGAGGGCAAGAAGAGTGT |
|  | SmKS-R | CTCCTGTTTGGTCGTTGAGAAGAATA |
